# Machine learning in knee arthroplasty: specific data are key—a systematic review

**DOI:** 10.1007/s00167-021-06848-6

**Published:** 2022-01-10

**Authors:** Florian Hinterwimmer, Igor Lazic, Christian Suren, Michael T. Hirschmann, Florian Pohlig, Daniel Rueckert, Rainer Burgkart, Rüdiger von Eisenhart-Rothe

**Affiliations:** 1 Department of Orthopaedics and Sports Orthopaedics, Klinikum Rechts Der Isar, School of Medicine, Technical University of Munich, Ismaninger Str. 22, 81675 München, Germany; 2 Institute for AI and Informatics in Medicine, Technical University of Munich, Munich, Germany; 3 Department of Orthopaedic Surgery and Traumatology-Liestal, Kantonsspital Baselland, Bruderholz, Laufen, Switzerland

**Keywords:** Artificial intelligence, Machine learning, Knee surgery, Total knee arthroplasty, Knee arthroscopy, Supervised learning

## Abstract

**Purpose:**

Artificial intelligence (AI) in healthcare is rapidly growing and offers novel options of data analysis. Machine learning (ML) represents a distinct application of AI, which is capable of generating predictions and has already been tested in different medical specialties with various approaches such as diagnostic applications, cost predictions or identification of risk factors. In orthopaedics, this technology has only recently been introduced and the literature on ML in knee arthroplasty is scarce. In this review, we aim to investigate which predictions are already feasible using ML models in knee arthroplasty to identify prerequisites for the effective use of this novel approach. For this reason, we conducted a systematic review of ML algorithms for outcome prediction in knee arthroplasty.

**Methods:**

A comprehensive search of PubMed, Medline database and the Cochrane Library was conducted to find ML applications for knee arthroplasty. All relevant articles were systematically retrieved and evaluated by an orthopaedic surgeon and a data scientist on the basis of the PRISMA statement. The search strategy yielded 225 articles of which 19 were finally assessed as eligible. A modified Coleman Methodology Score (mCMS) was applied to account for a methodological evaluation.

**Results:**

The studies presented in this review demonstrated fair to good results (AUC median 0.76/range 0.57–0.98), while heterogeneous prediction models were analysed: complications (6), costs (4), functional outcome (3), revision (2), postoperative satisfaction (2), surgical technique (1) and biomechanical properties (1) were investigated. The median mCMS was 65 (range 40–80) points.

**Conclusion:**

The prediction of distinct outcomes with ML models applying specific data is already feasible; however, the prediction of more complex outcomes is still inaccurate. Registry data on knee arthroplasty have not been fully analysed yet so that specific parameters have not been sufficiently evaluated. The inclusion of specific input data as well as the collaboration of orthopaedic surgeons and data scientists are essential prerequisites to fully utilize the capacity of ML in knee arthroplasty. Future studies should investigate prospective data with specific and longitudinally recorded parameters.

**Level of evidence:**

III.

## Introduction

Artificial intelligence (AI) has already led to tremendous advancements in the aviation and automobile industries and is now rapidly gaining importance in healthcare [1, 3, 18]. Machine learning (ML) represents a distinct application of AI which emerged from studies on pattern recognition and learning theory. ML describes algorithms for automatic and incremental function optimization which can be used to make predictions by detecting non-linear relationships in large data sets [1]. In a nutshell, ML algorithms are able to recognize correlations in data sets which subsequently permits predictions based on these “learned” patterns. The aspect of “learning” is achieved by automated weighting of distinct parameters in the mathematical model [1, 19]. Ever more data are being compiled digitally in healthcare while processing capacities are increasing rapidly so that various ML algorithms have already been tested in ophthalmology, dermatology, radiology, and cardiology [3, 5, 6, 18]. Especially in medical image analysis, ML algorithms were developed and validated which are capable to outperform medical specialists [17]. In orthopaedics, the number of published studies on machine learning has increased tenfold since 2010 [1]. ML has the potential to become a powerful tool for patient-specific decision making regarding surgical indications and predicting outcomes in orthopaedics. Although the application of ML is in a preliminary phase in orthopaedics, several studies report well-performing ML models for gait classification, fracture detection, exoprosthesis control, osteoarthritis detection, spine pathology detection and bone age assessment [19]. Extensive data are necessary for well-performing ML models. In this context, knee arthroplasty is highly suitable for ML analysis as multiple sources are available: national and international registries including follow-up data, patient characteristics, and patient-reported outcome measures after knee surgery from the last 40 years. At the same time, recently introduced enabling technologies such as navigated and robotic surgery offer novel, patient-specific data sets, and a vast amount of imaging data are available in digital form. Furthermore, various other clinical data may be suitable for ML models. Recently, an ML approach using registry and healthcare data sets to provide an adjusted payment model was demonstrated [23]. Overall, however, literature on ML in knee arthroplasty is heterogeneous and scarce.

ML comprises different theoretical approaches and represents a tool that must first be adapted for specific tasks in a given field such as knee arthroplasty. However, no standard approach for implementing ML algorithms in orthopaedics has been described nor established yet so that the question arises to which extent knee arthroplasty currently benefits from ML and which approaches might be promising. We hypothesized that successful ML algorithms in knee arthroplasty depend on arthroplasty-specific data and their reasonable use. Therefore, the collaboration between a data scientist and an orthopaedic surgeon is of utmost importance. Hence, we aim (1) to investigate which predictions are already feasible using ML models in knee arthroplasty and (2) to describe prerequisites for the effective use of this novel approach. For this reason, we conducted a systematic review of ML algorithms for outcome prediction in knee arthroplasty.

## Materials and methods

A systematic review of the literature was performed to identify machine learning algorithms in knee arthroplasty on the basis of the PRISMA statement. Studies meeting the following criteria were included in this review:

- Described methodology of a machine learning algorithm for data analysis in health or economic-related applications of knee arthroplasty.
- At least one predicted outcome variable by a supervised machine learning algorithm using tabular data.
- Written in English.

**Fig. 1:**
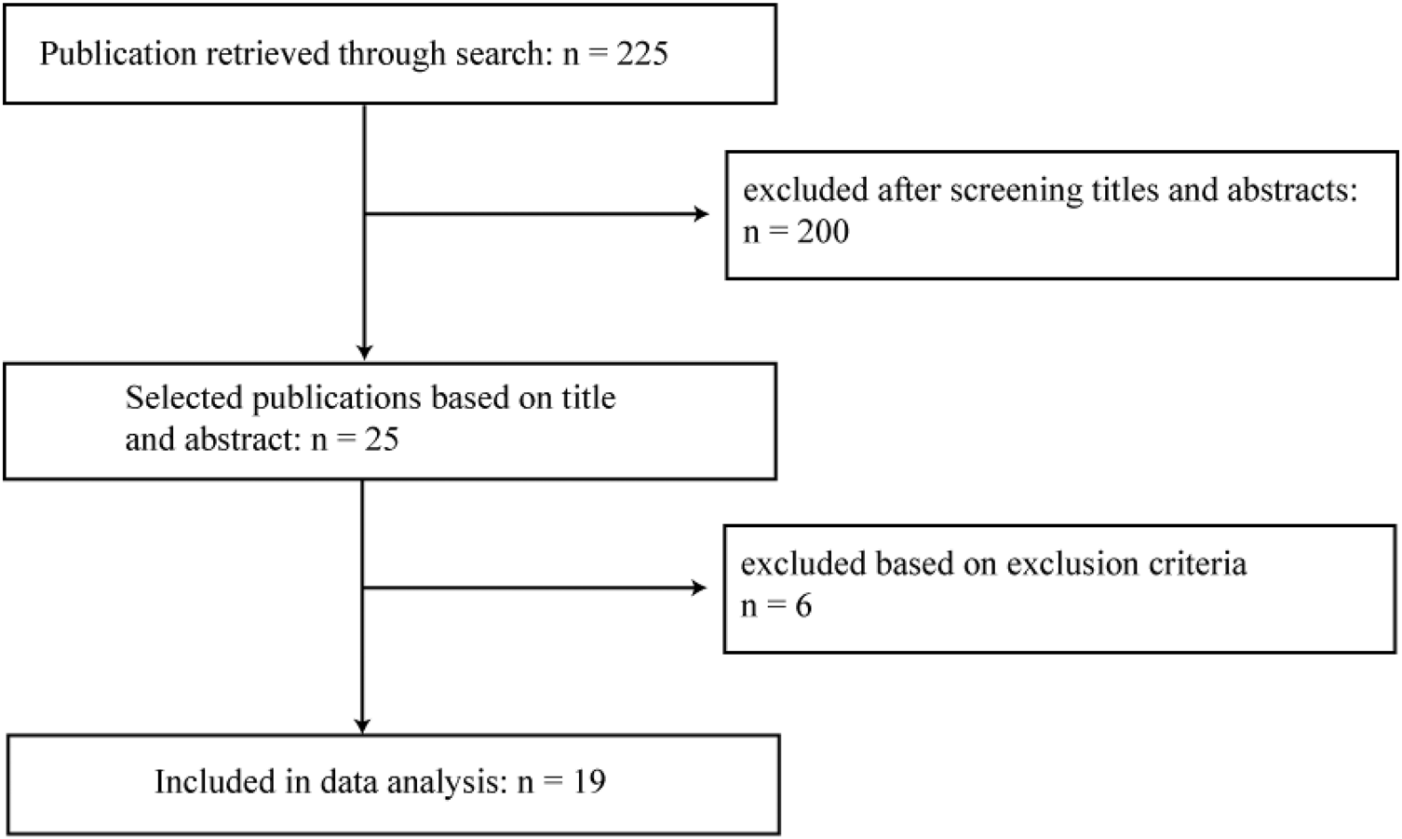
Flow diagram. The initial search through the PubMed and Medline database as well as Cochrane Library resulted in 225 publications (March 2021). After screening the titles and abstracts, 200 were excluded and 25 remained. After applying the exclusion criteria, another 6 were excluded and 19 remained for final investigation

In March 2021, a literature search through PubMed, Medline database and Cochrane Library was conducted. For the systematic search, the following search terms were used: “total knee arthroplasty” AND (artificial intelligence OR machine learning), “TKA” AND (artificial intelligence OR machine learning) from 1990 to 2021. The studies were screened and evaluated by an orthopaedic surgeon (I.L.) and a data scientist (F.H.) at our institution using the aforementioned selection criteria. The results were summarized and duplicates were discarded. The selection procedure is presented in Fig. 1. All articles were initially screened for relevance by title and abstract to assess the inclusion criteria. The two authors independently performed a careful reading of the studies and extracted the data. The following information was extracted from each article: author, year of publication, study design, follow-up, number of patients/cases, ML algorithm, metric, data screening, fine-tuning, mathematical and medical interpretation. For quality assessment, the Coleman Methodology criteria, which assess methodology using ten different aspects, were modified for the systematic review of machine learning algorithms in knee arthroplasty (Table 1). A score of 100 indicates that the study largely avoids chance, bias and other confounding factors. Hence, the modified Coleman Methodology score (mCMS) can be defined as excellent (85–100 points), good (70–84 points), fair (50–69 points), and poor (< 50 points). Both the orthopaedic surgeon and the data scientist scored the included studies giving a total mCMS between 0 and 100. In the event of divergent results, the data were double-checked and a consensus was reached. The level of evidence is level III.

**Table 1:** Modified Coleman methodology score

Part A—only one score to be given for each of the seven sections			
*1. Study size—number of patients (N)*		*2. Mean follow-up*	
*N* > 500	10	> 5 years	10
*N* 100–500	7	1–5 years	5
*N* 20–100	4	< 1 year, not stated, or unclear	0
*N* < 20 or not stated	0		
*4. Type of study*			
Multiple-outcome variables	10	Prospective cohort study	15
Single-outcome variable	5	Retrospective cohort study	10
		Experimental data set	5
*5. Number of input variables*		*6. Description of ML-approach*	
> 25	5	Technique stated with necessary details to repeat	10
10—25	3	Technique named without elaboration	5
< 10 or unclear	0	Not stated or unclear	0
*7. Fine-tuning of ML-model*			
Yes	5		
No	0		

Part B—scores may be given for each option in each of the three sections if applicable			
*1. Metrics*		*2. Data screening*	
Suitable metrics	5	Data processing elaborated and stated	5
More than one metric stated	5	Data source stated	5
External dataset for final evaluation	5		
*3. Mathematical and medical discussion*			
Metrics stated and elaborated in medical context	5		
Metrics statistically elaborated	5		

### Statistical analysis

Continuous data were reported as median values with the respective range. All analyses were performed using the statistical software IBM SPSS Statistics (version 22.0, Armonk, NY, IBM Corp.).

## Results

### Selection and methodical characteristics

The initial search resulted in 255 references and 19 articles met the inclusion criteria (Fig. 1). Articles from the years 2018 to 2021 were included. All 19 included studies addressed total knee arthroplasty (TKA). Fifteen articles were retrospective study designs, one study evaluated retrospective and prospective data and one further study analysed prospective data only. Two studies analysed an ML approach for outcome prediction using an experimental data set. The data set volume consisting of patients or cases ranged from 24 to 1,049,160 with a median of 86. The median follow-up time was 4.0 (1–18) years. The data sources were indicated in all studies. 11 of 19 studies derived their data exclusively from their local or regional healthcare management system. Three of 19 studies obtained their data from two administrative databases (National Inpatient Sample administrative database, New York Statewide Planning and Research Cooperative System administrative database). One study complemented their local database with data from a national registry (Korean Society of Nephrology registry). One study derived all their data from a national registry (Danish Knee Arthroplasty Registry). Another study evaluated data generated by a mobile application. All studies specified a data screening. The studies included a median of 13.5 (8;52) input parameters in their analysis. Fifteen different ML algorithms were described and four studies applied multiple approaches with different algorithms. Random Forest (RF) was applied in five studies, Gradient Boosting in three studies, LASSO in three studies, and Artificial Neural Network (ANN) in two studies, respectively. All other algorithms were applied once. 18 studies presented an outcome metric. With 16 of 19 studies, the area under the curve (AUC) was reported most frequently. 13 of these 16 studies reported AUCs with poor to fair results (< 0.8). In total, seven studies indicated a task-specific fine-tuning of the algorithm, including methodology such as data pre-processing, loss weighting or hyperparameter search. Three studies demonstrated an interpretation of metrics and outcomes from a mathematical as well as a medical point of view. The mCMS was calculated for each of the studies reviewed. The median mCMS of all studies was 65 points, ranging from 40 to 80 points. There was no significant relationship between methodology scores and metrics. However, only 6 out of 19 studies had a good mCMS (> 70 points). Of these six studies, five studies yielded fair to good results [AUC > 0.7/mean squared error (MSE) < 0.3], with the exception of one study with an AUC of 0.56–0.6. Methodological deficiencies were the lack of prospective studies, missing necessary details to repeat the ML model, no fine-tuning of the ML model and modest elaborations of the metrics in the mathematical and medical context. All further results are summarized in Tables 2, 3 and 4.

**Table 2:** Outcome variables and characteristics of the included studies

Prediction	Author	Year	Study design	Patient/case volume	Follow-up (yrs)	Outcome variable
Complications						
	Jo et al	2020	Retrospective	1686	6	Blood transfusion after TKA
	Katakam et al.	2020	Retrospective	12,542	18	Prolonged postoperative opioid prescriptions after TKA
	Ko et al.	2018	Retrospective	5757	7	End-stage renal disease after TKA
	Li et al.	2020	Retrospective	1826	1	Length of stay
	Navarro et al.	2019	Retrospective	141,446	7	Length of stay & inpatient costs
	Ramkumar, Karnuta et al.	2019	Retrospective	175,042	4	Length of stay & inpatient costs
Costs						
	Navarro et al.	2019	Retrospective	141,446	7	Length of stay & inpatient costs
	Ramkumar, Karnuta et al.	2019	Retrospective	175,042	4	Length of stay & inpatient costs
	Hyer et al.	2020	Retrospective	1,049,160	4	Super-utilizers = top 5%of health care users, responsible for 40% to 55%of all health care costs for TKA
	Karnuta et al.	2019	Retrospective	295,605	7	Inpatient procedural cost of Lower Extremity Arthroplasty
Functional outcome						
	Harris et al.	2020	Prospective	637	1	Knee Injury and Osteoarthritis Outcome Score (KOOS) after TKA
	Kluge et al.	2020	Retrospective	24	–	Spatio-temporal gait parameters after TKA
	Pua et al.	2021	Retrospective	4026	4	Walk time < = 15 min on six months postoperatively after TKA
Revision						
	El-Galaly et al.	2020	Retrospective	31,274	3	Revision within 2 years after TKA
	Shohat et al.	2020	Retrospective	1174	12	Revision after irrigation and debridement for PJI in THA and TKA
Postoperative satisfaction						
	Farooq et al.	2020	Retrospective Prospective	1325	5	Likert 5-point scale after TKA
	Kunze et al.	2018	Retrospective	430	2	Satisfaction—binary outcome 2 years after TKA
Surgical technique						
	Verstraete et al.	2020	Experimental	479	1	Optimal balanced TKA
Biomechanical properties						
	Rexwinkle et al.	2018	Experimental	6	–	Articular cartilage biomechanics

**Table 3:** Description of machine learning approaches of the included studies

Author	Year	Patient/case volume	Algorithm	Metric	Data screening	Fine tuning	Mathm. + medical interpretation	Modified Coleman Score
El-Galaly et al.	2020	31,274	LASSO, RF, Gradient Boosting, NN	AUC 0.57–0.6	Yes	Yes	Not specified	80
Farooq et al.	2020	1325	TreeNet	AUC 0.81	Yes	Not specified	Not specified	63
Harris et al.	2020	637	Logistic regression, LASSO	AUC 0.71–0.76	Yes	Not specified	Not specified	70
Hyer et al.	2020	1,049,160	Logic Forest	Not specified	Yes	Not specified	Not specified	58
Jo et al.	2020	1686	Gradient boosting	AUC 0.88	Yes	Not specified	Not specified	60
Karnuta et al.	2019	295,605	MLP, DenseNet	AUC 0.81	Yes	Yes	Not specified	78
Katakam et al.	2020	12,542	Stochastic gradient boosting	AUC 0.76	Yes	Not specified	Not specified	65
Kluge et al.	2020	24	Decision tree	Accuracy 0.89	Yes	Not specified	Not specified	49
Ko et al.	2018	5757	Gradient boosting	AUC 0.89	Yes	Yes	Not specified	70
Kunze et al.	2018	430	RF	AUC 0.77	Yes	Not specified	Not specified	58
Li et al.	2020	1826	XGBoost	AUC 0.74	Yes	Not specified	Not specified	58
Navarro et al.	2019	141,446	Naive Bayes	AUC 0.74–0.78	Yes	Not specified	Not specified	60
Navarro et al.	2019	141,446	Logistic regression	AUC 0.73–0.75	Yes	Yes	Not specified	75
Pua et al.	2021	4026	XGBoost, RF, LASSO, SuperLearner	AUC 0.7	Yes	Not specified	Not specified	68
Ramkumar, Haeberle et al.	2019	175,042	ANN	AUC 0.76–0.83	Yes	Not specified	Not specified	45
Ramkumar, Karnuta et al.	2019	175,042	ANN	MSE 0.21, 0.18	Yes	Yes	Yes	78
Shohat et al.	2020	1174	RF	AUC 0.74	Yes	Yes	Yes	68
Verstraete et al.	2020	479	RF, linear support vector machine, ANN	AUC 0.75–0.98	Yes	Yes	Yes	67
Rexwinkle et al.	2018	6	ANN	MSE 0.18	Not specified	Yes	Not specified	40

**Table 4:** Input variables of the included studies

Author	Year	Number of input variables (*n*)	Input variables	Data sources
El-Galaly et al.	2020	26	Sex, age, weight, height, BMI. observation year, revisions, Indications for TKA, Prior knee procedures, CCS, AKSS, coronal alignment, ap instability, mediolateral instability, walking distance, walking ability, stair-walking ability, need for a walking aid, choice of implant constraint, patella resurfacing, additional components, choice of fixation, use of intraoperative navigation, use of tourniquet, hospital knee volume, geographical region	Danish Knee Arthroplasty Registry
Farooq et al.	2020	15	Age, BMI, LOS, FU, generation, sex, ASA, surgeon, type of implant, PCL adressed, Depression, Inflammatory condition, preoperative narcotic use, Lumbar spine pain/surgery/disease, Tourniquet	Local database
Harris et al.	2020	28	Age, BMI, sex, race/ethnicity, marital status, education, employment status, CHF, Valvular disease, Peripheral vascular disease, Hypertension, Neurological disorders, CP, DM, Hypothyroidism, Renal failure, Liver disease, solid tumour without metastasis, Rheumatoid arthritis, weight loss, fluid and electrolyte disorders, deficiency anaemia, alcohol use disorder, drug use disorder, depression, AUDIT-C, PHQ, KOOS	Local database
Hyer et al.	2020	12	Age, sex, race, type of surgery, CCS, Elixhauser comorbidity score, Centers for Medicare & Medicaid Services–Hierarchical Condition Category, LOS, morbidity, readmission, mortality	Medicare inpatient and outpatient Standard Analytic Files
Jo et al.	2020	8	Tranexamic acid, Unilateral, Staged bilateral, Simultaneous bilateral, Platelet count, Age at surgery, Body weight, Hb	Local database
Karnuta et al.	2019	11	Age group, gender, ethnicity, race, APR-SOL, APR-ROM, Healthcare Research and Quality Clinical Classifications Software diagnosis code, type of admission, type of stay, discharge disposition, LOS	New York State-wide Planning and Research Cooperative System (SPARCS) administrative database
Katakam et al.	2020	39	Age, sex, race, ethnicity, marital status, disposition, Hb, WBC, platelets, creatinine, insurance status, neighborhood (zip code) characteristics, angiotensin converting enzyme inhibitor, angiotensin ii receptor blocker, antidepressant, beta-2-agonist, beta-blocker, benzodiazepine, gabapentin, immunosuppressant, NSAID, opioid, anti-psychotics, tobacco use, alcohol abuse, drug abuse, diabetes, renal failure, depression, psychoses, CHF, myocardial infarction, peripheral vascular disease, cerebrovascular accident, CP, arrhythmias, valvular disease, malignancy, liver disease	Local database
Kluge et al.	2020	8	Produced by the gait sensor: three-axis accelerometer, three-axis gyroscope, heel strike and toe off	Local database
Ko et al.	2018	18	Age, sex, BMI, ASA, type of anaesthesia, DM, types of surgery (unilateral, staged bilateral and simultaneous bilateral TKA), Blood urea nitrogen, creatinine, Hb, platelets, GFR, NSAID, antithrombotics, RAAS, diuretics, tranexamic acid	Local database, Korean Society of Nephrology registry
Kunze et al.	2018	15	Age, BMI, gender, preoperative opioid use, smoking history, DM, drug allergies, number of comorbid conditions, fibromyalgia/depression status, prior ipsilateral knee procedure not including a TKA, degree of flexion contracture of the operative knee, degree of knee flexion, preoperative patient-reported health state, KKS, KKS-F	Local database
Li et al.	2020	14	Age, race, gender, BMI, Hb, operation duration, history of smoking, DM, cerebrovascular accident, ischaemic heart disease, CHF, ASA, type of anaesthesia, creatinine	Local database
Navarro et al.	2019	8	Age group, CCS, ethnicity, gender, patient disposition, type of admission, APR-SOL, APR-ROM	New York State-wide Planning and Research Cooperative System (SPARCS) administrative database
PUA ET AL.	2021	25	Age, weight, height, BMI, race, sex, contralateral knee pain, hypertension, dyslipidemia, DM, adult recon specialist, caregiver available, education Level, gait aids, knee pain, depression, Anxiety, difficulty when climbing down stairs	Local database
Ramkumar, Haeberle et al.	2019	6	Step count, range of motion, KOOS, visual analogue scale, opioid consumption, home exercise program compliance	Mobile application database
Ramkumar, Karnuta et al.	2019	13	Age, gender, ethnicity, race, type of admission, APR-ROM, APR-SOL, number of associated chronic conditions and diagnoses, comorbidity status, whether the admission was on a weekend, hospital type, income quartile of the patient, transferred from an outside hospital	The OrthoMiDaS (Orthopedic Minimal Data Set) Episode of Care (OME) database, National Inpatient Sample (NIS) administrative database
Rexwinkle et al.	2018	12	Histological (cartilage structure, chondrocytes, proteoglycans, collagen, tidemark), mechanical (compressive stress relaxation), microbiological (tissue modulus, collagen fibre strength, tissue permeability) and proteomic (PIIANP, NO, and MMP-13)	Local database
Shohat et al.	2020	52	Timing in days (Acute postoperative/Acute haematogenous), age, sex, BMI, Smoking, Alcohol, Joint, Hypertension, Ischaemic heart disease, Heart failure, Oral anticoagulants, DM, CP, renal failure, malignancy, Liver cirrhosis, Rheumatoid arthritis, Immunosuppression, Index surgery was a revision, Index surgery used cemented prosthesis, indication for arthroplasty (osteoarthritis, rheumatoid arthritis, fracture, malignancy), wound leakage, skin necrosis, skin infection, fistula, fever, C-reactive protein, WBC, Positive blood cultures, Exchange of mobile component, MSSA, MRSA, Staphylococcus epidermidis, Streptococcus spp, Enterococcus spp, Escherichia coli, Enterobacter spp, Pseudomonas spp, Proteus spp, Candida spp, Polymicrobial	Local database
Verstraete et al.	2020	8	Intraoperative load and alignment readings by surgical navigation and smart tibial trial components	Local database

*BMI* Body Mass Index, *ASA* American Society of Anesthesiologist Physical Status, *CCS* Charlson comorbidity score, *AKSS* American Knee Society Score, *LOS* length of stay, *FU* Follow-up, *PCL* posterior cruciate ligament, *CHF* congestive heart failure, *CP* Chronic pulmonary disease, *DM* Diabetes mellitus, *AUDIT-C* Alcohol Use Disorders Identification Test Consumption, *PHQ* Patient Health Questionnaire, *KOOS* Knee Injury and Osteoarthritis Outcome Score, *Hb* Haemoglobin, *APR-SOL* All Patient Refined severity of illness, *APR-ROM* All Patient Refined risk of mortality, *WBC* white blood cells, *KSS* Knee Society Score, *KSS-F* KSS-Function

### Prediction of complications

Six studies evaluated ML algorithms predicting complications after the implantation of TKA [9, 12, 14, 16, 20, 23]. Three studies focused on the prediction of the length of stay [16, 20, 23]. Of these three studies, Gradient Boosting, Naive Bayes and an ANN were applied, resulting in a mean AUC of 0.78 (0.74; 0.83). They evaluated 8, 13 and 14 input variables, respectively. A single study evaluated a Gradient Boosting with 18 input variables for the prediction of end-stage renal disease after TKA, resulting in an AUC of 0.89 [14]. Another study analysed a Gradient Boosting with eight input variables for the prediction of postoperative blood transfusions after TKA, resulting in an AUC of 0.88 [9]. A further study evaluated a Stochastic Gradient Boosting with 39 input variables for the prediction of postoperative opioid prescriptions after TKA, resulting in an AUC of 0.76 [12].

### Prediction of costs

Four studies evaluated ML algorithms predicting the costs related to the implantation of TKA [8, 10, 20, 23]. Three studies evaluated the inpatient costs using MLP, DenseNet, Naive Bayes and an ANN with 13, 8 and 11 input variables, respectively. This resulted in a mean AUC of 0.79 (0.74; 0.81) [11, 20, 23]. Another study evaluated a Logic Forest algorithm with 11 input variables for the prediction of the most expensive 5% of health care users in the year following elective surgery (super-utiliser) [8]. TKA comprised 50% of 1,049,160 analysed surgeries. No metric was specified.

### Prediction of functional outcome

Three studies evaluated ML algorithms predicting the functional outcome after the implantation of TKA [7, 13, 21]. One study prospectively evaluated a logistic regression and least absolute shrinkage and selection operator (LASSO) with 28 input variables for the prediction of the Knee Injury and Osteoarthritis Outcome Score (KOOS) one year after TKA, resulting in an AUC of 0.71 to 0.76 [7]. Another study analysed a decision tree with eight preoperative input variables provided by a gait sensor for the prediction of gait parameters after TKA, yielding an accuracy of 0.89 [13]. A further study evaluated four different ML approaches (XGBoost, RF, LASSO and SuperLearner) with 25 input variables for the prediction of severe walking limitation after TKA. XGBoost yielded the most promising results with an AUC of 0.7 [21].

### Prediction of revisions

Two studies evaluated ML algorithms predicting revisions after the implantation of TKA [2, 25]. The first study evaluated four different ML approaches (LASSO, RF, Gradient Boosting, ANN) with 26 input variables for the prediction of revisions within two years after primary TKA, resulting in an AUC of 0.56–0.6 [2]. The second study analysed an RF with 52 input variables for the prediction of revision after irrigation and debridement for PJI in TKA, yielding an AUC of 0.74 [25].

### Prediction of postoperative satisfaction

Two studies evaluated ML algorithms predicting the postoperative satisfaction after the implantation of TKA [4, 15]. The first study evaluated a TreeNet with 15 input variables for the prediction of postoperative satisfaction after TKA with minimum 1-year follow-up using a 5-point Likert scale. This approach resulted in an AUC of 0.81 [4]. The second study analysed an RF with 15 input variables for the prediction of postoperative satisfaction 2 years after TKA using a binary outcome. This approach yielded an AUC of 0.77 [15].

### Prediction of optimal surgical technique

A single study evaluated the numerical data created in the surgical process to assess balance and alignment during TKA [26]. The authors suggested a decision process for an ideally balanced TKA. Three different ML approaches (RF, Support Vector Machine, ANN) were applied and resulted in AUCs of 0.75–0.81.

## Discussion

The most important finding of the present review is that outcome prediction using ML models applying arthroplasty-specific data has already been successfully performed. Although ML applications for knee arthroplasty only gained particular popularity in the past years with all articles included in this review being from 2018 or more recent, several studies already demonstrated the high potential of ML. Exact study questions with well-described complications like renal failure or postoperative blood transfusions can already be particularly well-predicted [9, 14]. However, more complex and general predictions like revisions, which can have a variety of different causes, are more difficult to estimate [2]. Interestingly, registry data on knee arthroplasty were only scantly evaluated for ML analysis. In data science theory, the sheer quantity of patients and input parameters is of crucial importance. Registries include vast information with patient-related data and potentially specific patterns that are suitable for ML analyses. However, most studies (13/19) relied on local databases and only six studies applied registry data—including only two different medical registries. Multiple national registries on knee arthroplasty are being administered that contain relevant information from the last decades on this topic. Future studies for ML in knee arthroplasty should definitely investigate the amounts of data within knee arthroplasty registries.

A further finding of this review is that we could not demonstrate a correlation between the amount of input parameters or patients and the outcome metrics. From a data science perspective, the tabular form of clinical information is not well suited for ML analysis as it requires a tremendous amount of patients and various parameters to generate complex data sets. Six studies included more than 100,000 patients; however, ML is proficient to process substantially larger amounts of data. Image data contain a higher information density and is, therefore, more commonly applied to ML applications. However, the low data volume is only in part an explanation for poor outcomes as several studies yielded very accurate predictions, especially in confined, local data sets.

While the quantity of data remains critical to allow for repeating patterns, the data quality is likewise of high importance for ML algorithms: ML can only reveal patterns that can actually be derived from the data. While Karnuta et al. fed their algorithm with tabular data from 295,605 total knee arthroplasty and total hip arthroplasty patients, Rexwinkle et al. retrieved tissue samples from only 6 osteoarthritis patients in order to apply biomarker analysis as well as biomechanical testing [10, 24]. The latter study was based on much fewer cases, still the complexity of the study and specificity of the data allowed for ML application with promising results. We therefore assume that the inclusion of specific parameters is of paramount importance, especially if tabular data are applied for ML analysis.

Just as specific data are relevant, a multitude of irrelevant parameters may negatively impact the performance of ML models. Although an increased data width with the use of all accessible parameters may be beneficial in theory, such randomness may rather hinder the full potential of the ML algorithm within the limited scope of tabular data used so far for ML in knee arthroplasty. Especially in studies with a low proportion of subject-specific parameters, the input of parameters, which were chosen simply because they were available, may be overrated and confounding. Hence, the selection of data for ML applications is anything but trivial. In this review, only four studies included subject-specific parameters such as the administration of tranexamic acid for the prediction of postoperative blood transfusion after TKA. Therefore, further studies to investigate arthroplasty-specific parameters are encouraged.

Identifying and applying such specific parameters in knee arthroplasty is difficult, though. Currently, the parameter selection of ML approaches in orthopaedics is based on (1) known risk factors and clinical experience and (2)—to a significant part—on the accessibility of parameters in existing databases. Therefore, most studies apply administrative data including basic patient demographics. However, the data architectures of local healthcare management systems and registries were not constructed for ML evaluations. These data sources do not necessarily contain fully detailed and prospectively linked patient data, which, however, would be essential for predictions. In addition, most studies were retrospective so that the data architecture for the ML analysis had to be constructed post hoc. Hence, data screening and processing are inevitable if existing data sources should be utilized.

This retrospective approach requires the substantive discussion of the parameters to assure inclusion of relevant and exclusion misleading information, as well as the mere technical evaluation of the data with regard to incorrect inputs and missing data points. Further steps for enhancing the data set, such as data cleaning or completing a data set are recommended from a data science perspective. This data processing requires extensive human assessment and the coherent thinking of a medical professional and data scientist to identify relevant and applicable information. It has to be performed mostly by hand, which makes it time-consuming and futile. The work related to data screening and processing is not to be neglected: it dramatically slows down the potential progress that can be made using ML in knee arthroplasty. To avoid such an extensive data processing, we assume that prospective databases with an appropriate data architecture for ML analysis have to be established in the long term. Currently, we are unaware of a comprehensive and completed data integration of knee joint-specific data for AI applications. Besides the already discussed importance of the quality and amount of input data, the ML model itself has a significant impact on the outcome prediction. ML does not describe a single specific algorithm, but rather contains a variety of approaches which have to be modified to the addressed issue and data set. A wide range of algorithms was used in the studies discussed in this review. The most popular algorithm was an RF, which is closely related to decision trees and XGBoost. This may be attributed to the considerable easy implementation, application and stable results over different kinds of data sets. However, more sophisticated algorithms have been developed exploiting the potential of today’s data as well as hardware even more. With a considerable amount of data, even deep learning algorithms can be feasible. Ramkumar et al. demonstrated how to use an ANN for, e.g. length of stay prediction of 175,042 TKA cases using 15 preoperative variables [22]. To achieve the best results, the focus of most studies relied on testing various machine learning algorithms and picking the one with the highest metric value as final algorithm. However, an exclusive measurement of performance using only one metric can be misleading so that several metrics depending on the task at hand need to be examined. Metrics which weigh false-positive and false-negative predictions, such as sensitivity, specificity or Dice Score, are often more meaningful. Furthermore, prediction models based on ML algorithms are not explanatory models [7]. Therefore, the outcome metrics need to be elaborated to understand its use in the medical context. In this review, 16 of 19 studies did not extensively discuss the results in the mathematical or in the medical setting. For future investigations, we consider the cooperative analysis by a medical professional and a data scientist of ML models in knee arthroplasty as mandatory.

**Fig. 2:**
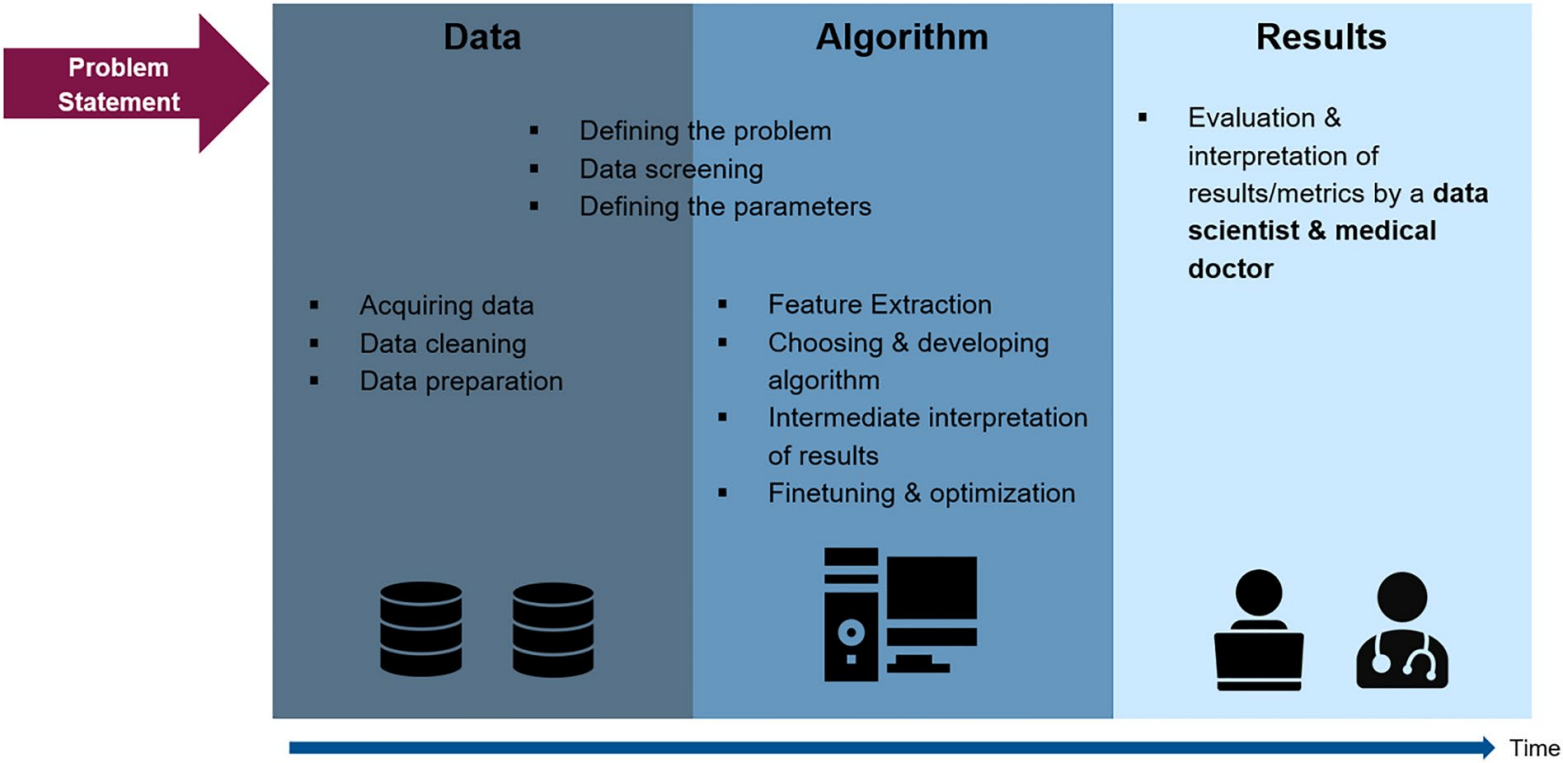
Overview of the development of a ML model in knee surgery. After defining the problem statement, the algorithm development consists of three main pillars: **a** data, **b** algorithm, **c** results. In step **a**, the dataset has to be established and prepared in a manner that it is qualitatively and quantitatively feasible for ML algorithms. In step **b**, an algorithm has to be chosen or developed and fine-tuned for the specific problem at hand. In step **c**, the results have to be evaluated by a computer scientist in collaboration with an orthopaedic surgeon. If the results are not yet satisfying, steps **b** and **c** can be iterated several times

Only few studies managed to increase the performance of their ML models through regularization or random search of hyperparameters in order to obtain more meaningful results [2]. For deep learning algorithms, multiple techniques, i.e., regularization and data augmentation, loss weighting, hyperparameter search or data simulation, are commonly applied to boost performance. In machine learning, these techniques are used not as frequently while still being possible most of the time. A hyperparameter search for instance contains significant potential and is easily applicable with today’s hardware capacity. Another underrated example for performance increase are methods to tackle class imbalance such as loss weighting. While a hyperparameter search was applied once [2], none of the reviewed studies indicated the use of any kind of loss weighting. From data science perspective, numerous modifications of the ML models in knee arthroplasty have not been implemented and tested yet. Especially for limited data sets, fine-tuning of ML models is crucial to avoid overfitting. Figure 2 depicts an overview of the development of an ML model in knee joint surgery based on this discussion. This review has several limitations. All studies on ML in knee arthroplasty were not presumed to be included. Numerous studies from the field of medical informatics examined big data that included data sets related to knee arthroplasty. However, we evaluated specific studies which focused on outcome prediction in knee arthroplasty from the medical perspective. This may have disregarded other studies using promising ML approaches in orthopaedics with other purposes. Especially imaging data are suitable for ML models, but those are mostly utilized in ML models for diagnostic applications. We deliberately did not evaluate ML algorithms using imaging data for that reason.

## Conclusion

In conclusion, the prediction of distinct outcomes with ML models applying specific data is already feasible in knee arthroplasty. However, the prediction of more complex and general outcomes is still inaccurate. Currently, the benefits for the clinical application may be small, but—from a data science perspective—the possibilities are not yet being fully utilized. To date, specific parameters of knee arthroplasty have not been adequately evaluated and large data volumes gathered in registries have not been fully considered nor analysed. The inclusion of specific input data as well as the collaborative development, modification and evaluation of ML models and its data by an orthopaedic surgeon and data scientist are essential prerequisites to fully utilize the capacity of ML in knee arthroplasty. Predictions suitable for clinical application must be built on solid data structures. We consider that a data architecture specifically for ML with prospective data is necessary to allow for more accurate and complex predictions. Future studies should, therefore, necessarily examine large-scale data with specific and longitudinally recorded parameters.
